# Microbial community dynamics in an ANAMMOX reactor for piggery wastewater treatment with startup, raising nitrogen load, and stable performance

**DOI:** 10.1186/s13568-018-0686-0

**Published:** 2018-10-01

**Authors:** Qiang Huang, Wei-Li Du, Li-Li Miao, Ying Liu, Zhi-Pei Liu

**Affiliations:** 1 State Key Laboratory of Microbial Resources, Institute of Microbiology, Chinese Academy of Sciences, No. 1 West Beichen Road, Chaoyang District, Beijing, 100101 People’s Republic of China; 2 University of Chinese Academy of Sciences, Beijing, 100049 People’s Republic of China

**Keywords:** ANAMMOX, Anammox bacteria, Microbial community dynamics, Stepwise increasing load, Piggery wastewater

## Abstract

Bacterial community dynamics of the ANAMMOX reactor of an integrated “UASB + SHARON + ANAMMOX” system for treating piggery wastewater were investigated using the Illumina MiSeq method with samples obtained at ~ 2-week intervals during a 314-day period. With aerobic activated sludge as seeds and low content artificial wastewater (NH_4_^+^–N 50 mg/L; NO_2_^−^–N 55 mg/L) as influent for the ANAMMOX reactor, nitrogen removal was initially observed on day 38 with a removal rate 1.3 mg N L^−1^ day^−1^, and increased to 90.4 mg N L^−1^ day^−1^ on day 55 with almost complete removal of ammonia and nitrite, indicating a successful startup of the reactor. Increasing influent load stepwise to NH_4_^+^–N 272.7 mg/L/NO_2_^−^–N 300 mg/L, nitrogen removal rate increased gradually to 470 mg N L^−1^ day^−1^ on day 228, and maintained a stable level (~ 420 mg N L^−1^ day^−1^) following introduction of SHARON effluent since day 229. Correlation between microbial community dynamics and nitrogen removal capability was significant (*r *= 0.489, *p *< 0.001). Microbial community composition was determined by influent ammonia, influent nitrite, effluent nitrate and some undefined factors. Anammox bacteria, accounting for ~ 98.7% of *Planctomycetes*, became detectable (0.03% relative abundance) since day 38 and increased to 0.9% on day 58, well consistent with nitrogen removal performance of the reactor. Relative abundance of anammox bacteria gradually increased to 38.4% on day 140 with stepwise increased influent load; decreased to 0.4% on day 169 because of nitrite inhibition; increased to 19.24% on day 233 when the influent load was dropped; kept at ~ 9.0% with SHARON effluent used as influent and dropped to 3.3% finally. Anammox bacteria, only *Candidatus Brocadia* and *Ca*. *Kuenenia* detected, were the most abundant at genus level. *Ca*. *Brocadia* related taxa were enriched firstly under low load and detectable during the entire experimental period. Three main groups represented by *Ca*. *Brocadia* related OTUs were enriched or eliminated at different loads, but *Ca*. *Kuenenia* related taxa were enriched only under high load (NO_2_^−^–N > 300 mg/L), suggesting their different niches and application for different loads. These findings improve the understanding of relationships among microbial community/functional taxa, running parameters and reactor performance, and will be useful in optimizing running parameters for rapid startup and high, stable efficiency.

**Electronic supplementary material:**

The online version of this article (10.1186/s13568-018-0686-0) contains supplementary material, which is available to authorized users.

## Introduction

Ammonia pollution from pig farm has steadily increased worldwide in recent decades and presents serious environmental problems (Ali et al. 2013). Ammonia and chemical oxygen demand (COD) are the two main and high content pollutants in piggery wastewater (Bernet et al. 2000; Zhu et al. 2013). Anaerobic digestion methods, e.g., upflow anaerobic sludge bed (UASB) reactor, have been widely used to eliminate COD coupled with biogas production (Hashimoto 1983). Conventional nitrification–denitrification methods require large amount of energy and organic materials, resulting in high operational cost and limited application (Bernet et al. 1996; Boiran et al. 1996).

The anaerobic ammonium oxidation process (ANAMMOX), a more recently developed approach for nitrogen removal from wastewater, has the advantages of high efficiency, low sludge production, and no organic material requirement (Strous et al. 1999). Application of ANAMMOX for treatment of ammonia-rich wastewater reduced operational costs by ~ 90% (Jetten et al. 2001). Anammox bacteria, members of *Planctomycetes*, oxidize ammonia with nitrite as electron acceptor to produce dinitrogen gas (N_2_) (Strous et al. 1999; van de Graaf et al. 1995). In order to satisfy ANAMMOX reactor, a half partial nitrification process, e.g. a single reactor for high activity ammonium removal over nitrite (SHARON), was necessary to convert ~ 50% of ammonia to nitrite. Combined process SHARON–ANAMMOX had been applied for piggery wastewater treatment, but the nitrogen removal efficiency was not ideal due to high organic content, which would seriously inhibite anammox bacteria (Hwang et al. 2005; Jin et al. 2012; Tang et al. 2010; Yamamoto et al. 2008).

In addition to anammox bacteria, heterotrophic bacteria, e.g. *Proteobacteria*, *Chloroflexi*, *Chlorobi*, *Bacteroidetes*, and *Acidobacteria*, also played important roles in ANAMMOX bioreactor (Chen et al. 2017; Hwang et al. 2005; Lawson et al. 2017; Suto et al. 2017). Microbial community compositions in a bioreactor directly determined the reactor efficiency (Cho et al. 2010; Finlay et al. 1997), and would shift to adapt to those environmental changes, including organic materials, substrates (nitrite, ammonia), salinity, and running parameters [dissolved oxygen (DO), pH, temperature] (Egli et al. 2001; Isaka et al. 2007; Jin et al. 2012). Most studies to date have investigated the microbial communities in ANAMMOX reactors, especially for piggery wastewater treatment (Chen et al. 2017; Hwang et al. 2005; Suto et al. 2017). However, those studies focused on microbial communities at a single time point, this did not permit evaluation of microbial community dynamics, mutual interactions among anammox bacteria and other microorganisms, or effects of environmental factors on microbial communities in reactors. Evaluation of microbial community changes in relation to environmental factors reveals relationships between community dynamics and micro-ecosystem functions (Finlay et al. 1997), and is useful for achieving quick startup, running parameter optimization, and stably efficient maintenance of ANAMMOX reactors. It is highly desirable to examine microbial community dynamics over the entire running period, in order to observe relationships among these dynamics, reactor performance, and environmental factors.

We recently constructed an integrated system termed “UASB + SHARON + ANAMMOX” for treating piggery wastewater. Performance of a laboratory-scale system was stable and efficient (MS submitted). In the present study, we used the Illumina MiSeq method to investigate microbial community dynamics of the ANAMMOX reactor of this integrated system with samples obtained at ~ 2-week intervals during a 314-day period. Correlations were examined between community dynamics and nitrogen removal efficiency during startup, acclimation period, introduction of SHARON effluent and stable/efficient treatment period. Environmental factors affecting the community dynamics were evaluated. Changes of anammox bacterial compositions were also elucidated during the entire running time. Our findings will be useful in comprehension of relationships among microbial community composition, nitrogen removal efficiency and running parameters, and help optimizing running parameters for rapid startup and stable performance of ANAMMOX reactors in practical application.

## Materials and methods

### ANAMMOX reactor and running parameters

A laboratory-scale “UASB + SHARON + ANAMMOX” system (Additional file 1: Fig. S1; Table S1) was constructed for experimental treatment of piggery wastewater (COD 5500–8500 mg/L/NH_4_^+^–N 500–1500 mg/L) obtained from an animal husbandry facility in Changping District, Beijing, China. The three reactors of the system were started up separately, and integrated on day 229. The purpose of the third reactor (ANAMMOX) was to remove nitrogen compounds (mainly ammonia and nitrite) present in effluent from the second reactor (SHARON). The ANAMMOX reactor was constructed of plexiglass [poly(methyl methacrylate)] with height 1400 mm, diameter 140 mm, and effective volume 13.3 L. Activated sludge obtained from the aeration tank of a wastewater treatment plant was used as inoculum, with filling ratio 30% (v/v) for startup. Concentrations of volatile solids (VS) and suspended solids (SS) in seed sludge were 3500 and 4870 mg/L, respectively. ANAMMOX temperature was maintained at 31–32 °C by a water jacket, with hydraulic retention time (HRT) 26.6 h. The reaction was started up with artificial wastewater (NH_4_Cl, NaNO_2_, NaHCO_3_, and Na_2_HPO_4_·12H_2_O; 7:10:2:2 w/w) having initially low nitrogen load (NH_4_^+^–N 50 mg/L/NO_2_^−^–N 55 mg/L), and increasing gradually to NH_4_^+^–N 300 mg/L/NO_2_^−^–N 330 mg/L. On day 229, artificial wastewater was replaced by SHARON effluent. DO and pH were not controlled.

### Sludge samples and DNA extraction

Sludge samples (21 in total) were collected from the bottom (activated sludge assembled at the bottom of bioreactor) of the ANAMMOX reactor at ~ 2-week intervals during the entire running period (314 days). Six SHARON reactor samples [obtained during days 220–290 in aeration status (Du et al. 2016)] were used as references.

Total genomic DNA was extracted from each sample (~ 0.5 g) using a PowerSoil DNA isolation kit (MO BIO Laboratories; Shenzhen, China) as per the manufacturer’s instructions, and stored at − 80 °C.

### Illumina MiSeq sequencing analysis of 16S rRNA gene amplicons

Bacterial communities of the 21 ANAMMOX samples and six SHARON samples were analyzed. The V3–V4 hypervariable region of bacterial 16S rRNA gene was amplified using primer set 338F/806R, and 468-bp fragments were obtained and subjected to sequencing/analysis on the Illumina MiSeq PE300 platform. Raw data were processed using the Quantitative Insights Into Microbial Ecology (QIIME v. 1.8.0) toolkit (Caporaso et al. 2010). Chimeric sequences were checked and filtered using UCHIME (Edgar et al. 2011). Quality reads were clustered into operational taxonomic units (OTUs) with 97% sequence similarity cutoff using UPARSE (Edgar & Robert, 2013). Representative sequence of each OTU was selected for taxonomic assignment using the Greengenes database, v. 13-8 (Wang et al. 2007). For all OTU-based analyses, sequence number was normalized prior to statistical analysis to the smallest sample size. QIIME was used to create Bray–Curtis distance metrics and *α*-diversity indexes, including ACE, Chao 1 richness estimation, Shannon, Simpson, and Good’s coverage. The analyzed sequences were deposited in Sequence Read Archive (SRA) database under accession number SRP108925.

### Analytical methods

NH_4_^+^–N, NO_2_^−^–N, and NO_3_^−^–N were determined using a water quality analyzer (Aquakem 600, Thermo Fisher Scientific). DO was measured with a DO meter (model JPSJ-605; Shanghai Precision & Scientific Instrument Co.; Shanghai, China). pH was measured with a pH meter (model PB-10; Sartorius; Germany).

### Statistical analysis

Patterns of microbial community dynamics in the ANAMMOX reactor during the entire running period were evaluated by Principal Coordinates Analysis (PCoA) based on Bray–Curtis distance (Gauch and Hugh 1973). Correlations between community dynamics and environmental factors were evaluated by redundancy analysis (RDA). The contribution of environmental factors in driving community dynamics was assessed using variation partitioning analysis (VPA). Correlations between community dynamics and nitrogen removal capacity were assessed by Mantel test. Pearson’s test was used to evaluate correlations between environmental factors and major phyla, and α-diversities. The above analyses were performed using the R software program (v. 3.2.1; http://www.r-project.org). Phylogenetic trees were constructed using the MEGA 6.0 software program (Tamura et al. 2013), based on representative sequence for each OTU, by neighbor-joining (NJ) method with bootstrap values calculated from 1000 replications.

## Results

### Performance of ANAMMOX reactor

**Fig. 1:**
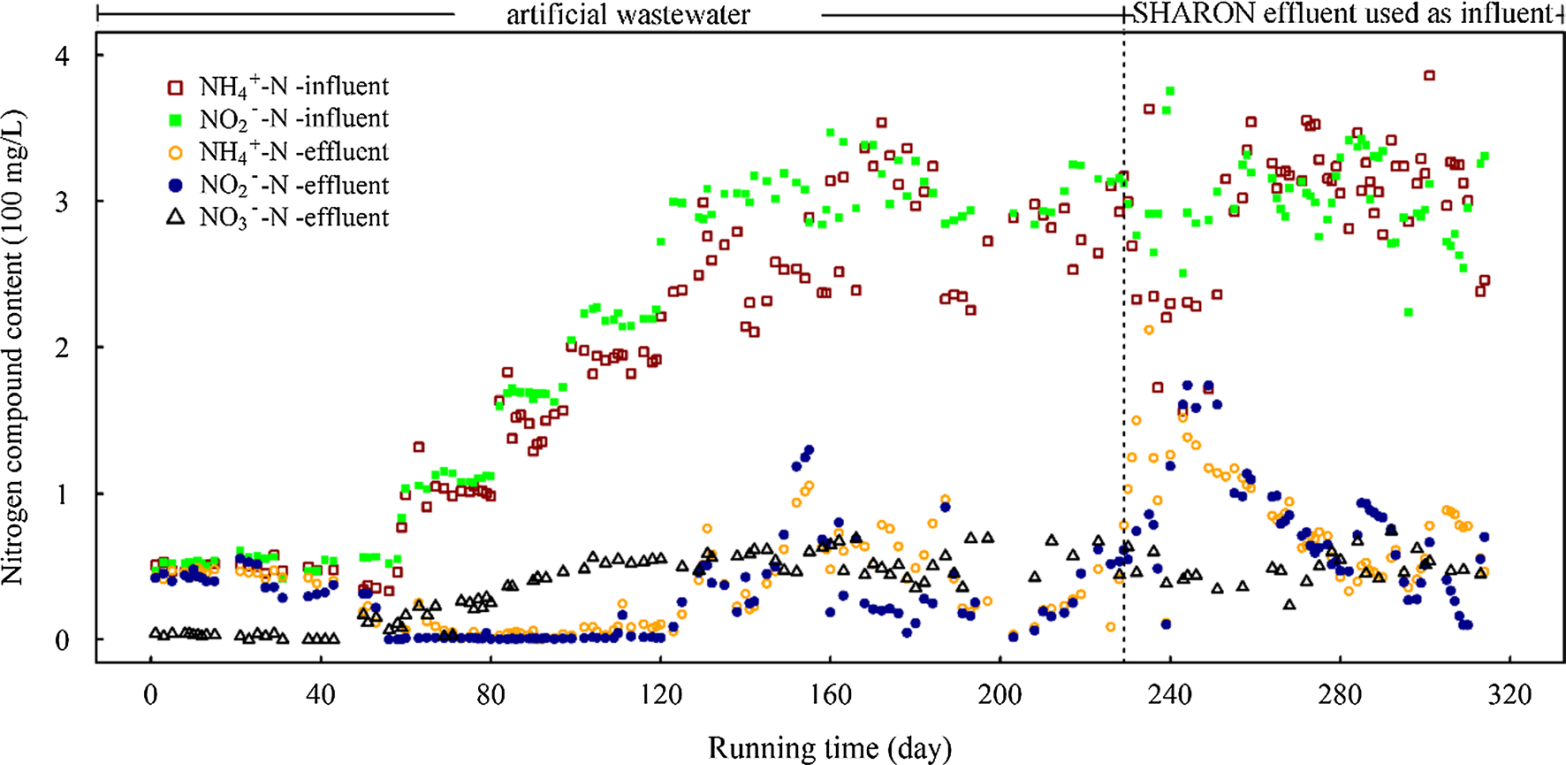
Performance of ANAMMOX reactor during entire running time. Dotted line: day 229, when SHARON effluent was introduced as ANAMMOX influent

Low influent load (NH_4_^+^–N 50 mg/L/NO_2_^−^–N 55 mg/L) was used for startup of ANAMMOX reactor. Effluent ammonia and nitrite levels showed no significant decrease through day 1 to day 37, then nitrogen removal was initially observed since day 38 with a removal rate of 1.3 mg N L^−1^day^−1^. Effluent ammonia and nitrite level dropped rapidly to undetectable level by day 55, and nitrogen removal rate reached to 90.4 mg N L^−1^day^−1^ (Fig. 1), indicating the successful startup. After day 59, influent load was increased gradually to NH_4_^+^–N 200 mg/L/NO_2_^−^–N 220 mg/L by day 120, during which time nitrate increased gradually to ~ 50 mg/L NO_3_^−^–N but no ammonia or nitrite was present in effluent (Fig. 1). Influent load was then increased gradually to NH_4_^+^–N 272.7 mg/L/NO_2_^−^–N 300 mg/L from day 130 to 160, with accompanying increases in effluent to NH_4_^+^–N ~ 60 mg/L, NO_2_^−^–N ~ 40 mg/L, and NO_3_^−^–N ~ 60 mg/L. From day 161 to 187, influent load was increased to NH_4_^+^–N 300 mg/L/NO_2_^−^–N 330 mg/L, accompanied by increase of NH_4_^+^–N in effluent to ~ 80 mg/L, but no notable increase of nitrite or nitrate. Nitrogen removal efficiency improved when the influent load decreased to NH_4_^+^–N 272.7 mg/L and NO_2_^−^–N 300 mg/L. Stepwise increasing influent load, total nitrogen removal rate increased gradually to 470 mg N L^−1^ day^−1^ at day 228, and was maintained at this level thereafter, despite the reduction in efficiency at high load.

SHARON effluent (piggery wastewater following anaerobic digestion and partial nitrification; NH_4_^+^–N 297.54 ± 48.96 mg/L, NO_2_^−^–N 301.69 ± 28.51 mg/L, NO_3_^−^–N 3.32 ± 1.44 mg/L) was used as ANAMMOX influent on day 229. Effluent nitrogen levels increased rapidly to NH_4_^+^–N ~ 163.6 mg/L/NO_2_^−^–N ~ 186.4 mg/L/NO_3_^−^–N ~ 60 mg/L (Fig. 1). Total nitrogen removal rate declined to 212 mg N L^−1^ day^−1^ by day 233. After ~ 1 month acclimation, effluent nitrogen decreased gradually and was then steadily maintained at NH_4_^+^–N ~ 42.8 mg/L/NO_2_^−^–N ~ 52.4 mg/L/NO_3_^−^–N ~ 57.2 mg/L, with nitrogen removal rate maintained at ~ 420 mg N L^−1^ day^−1^ (Fig. 1).

### Illumina MiSeq sequencing results, and community structure/dynamics

For the 27 total samples (21 ANAMMOX, 6 SHARON), 974,987 high-quality sequences in total were obtained from 1163,298 sequences of raw data after sequence processing. Number of sequences of individual samples ranged from 25,958 (SBR-220) to 46,740 (AN-109). Sequences for all samples were standardized to 25,958 for further analysis. Greengenes Database core 16S rRNA reference sequences were used for analysis of taxonomic structure of microbial communities. In total, 46 phyla, 107 classes, 156 orders, 181 families, and 1471 OTUs (97% sequence similarity cutoff) were classified. OTU numbers of anammox samples ranged from 374 (AN-159) to 706 (AN-21) (Table 1). Good’s coverage estimates were all > 99% (Table 1), indicating that nearly all bacterial species were included. Detailed phylogenetic analyses for the annotated genera are presented in Fig. S2.

**Table 1:** OTU richness and diversity indices of bacterial communities in samples

Samples^a^	OTUs^b^	ACE	Chao1	Shannon	Simpson	Good’s coverage (%)
AN-1	642	736.4	778.2	6.87	0.98	99.53
AN-21	706	833.1	865.1	6.85	0.98	99.40
AN-38	691	805.2	822.9	6.52	0.96	99.45
AN-58	649	779.7	806.1	6.37	0.96	99.41
AN-80	531	672.3	664.4	5.92	0.96	99.47
AN-93	477	601.3	596.5	6.13	0.97	99.51
AN-109	512	624.4	621.4	6.45	0.98	99.51
AN-123	433	555.2	558.2	5.17	0.93	99.53
AN-140	378	494.7	474.3	4.68	0.89	99.58
AN-159	374	477.6	458.4	5.04	0.92	99.61
AN-169	704	795.9	831	6.99	0.98	99.51
AN-191	377	477.4	464.3	5.00	0.92	99.61
AN-205	404	553.1	537.2	5.20	0.94	99.51
AN-219	391	533.5	534.5	4.79	0.91	99.52
AN-233	690	855.0	851.3	6.25	0.96	99.30
AN-239	455	532.5	631.9	5.33	0.95	99.42
AN-256	442	577.1	575	5.29	0.95	99.49
AN-271	479	691.9	670.5	5.16	0.94	99.35
AN-285	482	656.6	680.2	5.21	0.94	99.38
AN-297	503	657.1	706.1	5.59	0.95	99.41
AN-310	484	669.1	645.0	5.27	0.95	99.38

^a^Numbers following “AN” in this column indicate sampling date (day)

^b^97% similarity cutoff

**Fig. 2:**
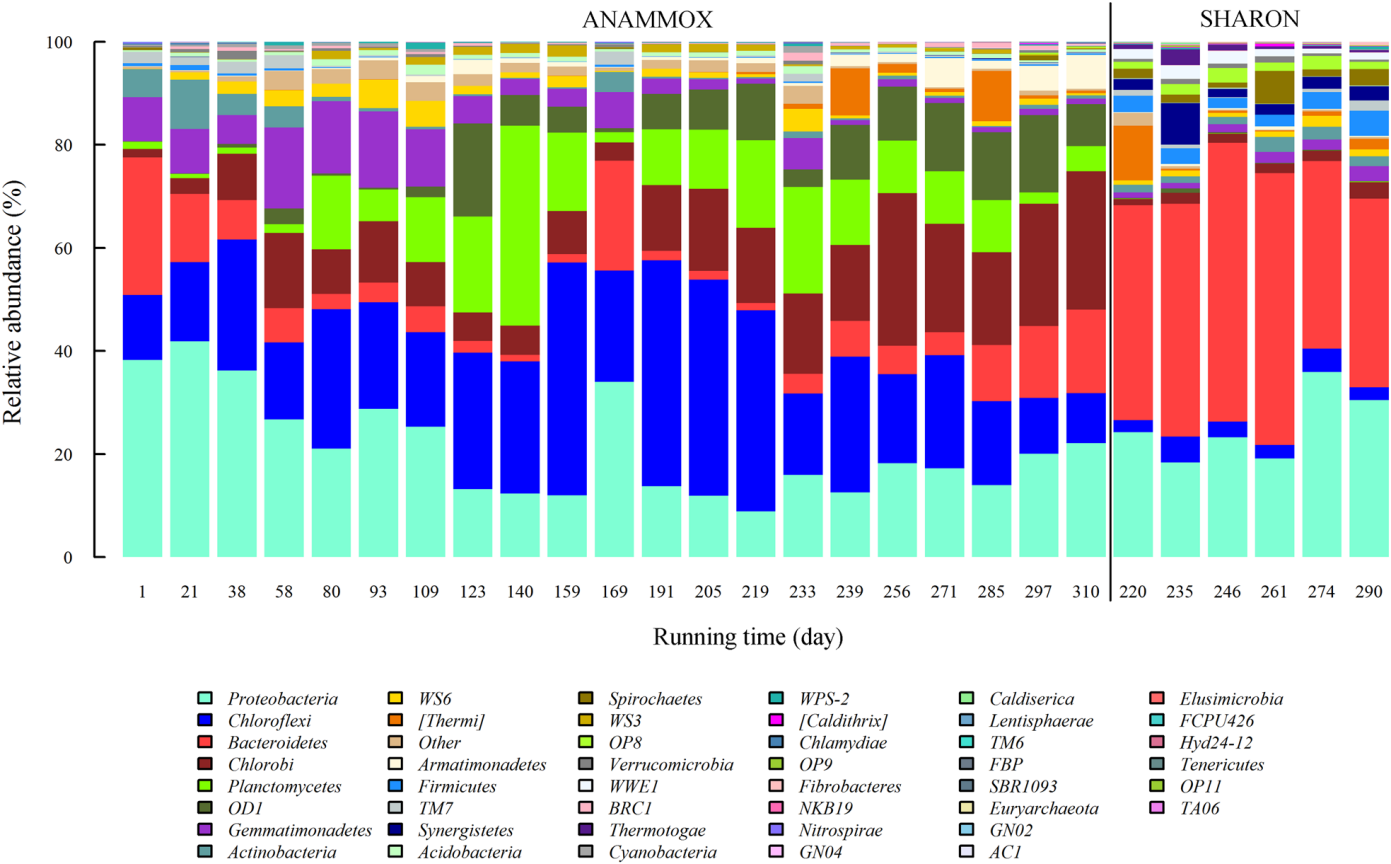
Relative abundance of phyla based on Illumina MiSeq bacterial 16S rRNA genes in ANAMMOX samples during the entire running period, and in SHARON samples when SHARON effluent was used as ANAMMOX influent

Relative abundance of phyla and dynamic changes in abundance in ANAMMOX reactor are shown in Fig. 2. There were six predominant phyla (*Proteobacteria*, *Chloroflexi*, *Chlorobi*, *Planctomycetes*, *Bacteroidetes*, *OD1*), each having average relative abundance > 5%, and sequences of these phyla jointly accounted for 67.6–91.8% of the total in each sample. Relative abundance of *Chloroflexi* increased gradually from 12.6% (day 1) to 45.5% (day 159), then declined to 9.7% (day 310). Abundance of *Proteobacteria* decreased from 38.3% (day 1) to 11.9% (day 159), increased sharply to 34.1% (day 169), then declined again to ~ 16.4% and stayed in that range. Abundance of *Chlorobi* rose from 1.7% (day 1) to 14.5% (day 58), declined gradually to 3.6% (day 169), then increased gradually to 26.9% (day 310). Abundance of *Planctomycetes* remained very low (~ 1.3%) during days 1–38, increased gradually to 38.9% (day 140), declined to 1.9% (day 169), rose again and stayed steady at ~ 13.00% until day 285, then finally dropped to ~ 4.8% and stayed there. Abundance of *Bacteroidetes* dropped from 26.6% (day 1) to 1.7% (day 159), increased sharply to 21.2% (day 169), dropped and stayed steady at ~ 1.7% until day 219, then increased gradually to 16.2% (day 310). Abundance of *OD1* was low (~ 0.9%) during days 1–109, increased sharply to 17.9% on day 123, declined to 0.8% on day 169, rose gradually to 15.3% on day 297, then fell to ~ 4.48% and stayed there. Pearson test showed that most of these relative abundance values showed significant correlations with nitrogen concentration (influent ammonia, influent nitrite, effluent nitrate) and nitrogen removal efficiency (Table 2). The two major phyla in SHARON reactor, accounting for 63.6–77.3% of total, were *Bacteroidetes* and *Proteobacteria* (Fig. 2). When SHARON effluent was introduced as ANAMMOX influent, bacteria in SHARON effluent had little or no effect on the anammox community; only a slight increase in *Bacteroidetes* abundance in later stages was observed (Fig. 2).

**Table 2:** Correlation (*R* value) of relative abundance of major phyla to nitrogen compounds

Phylum	NH_4_^+^–N		NO_2_^−^–N		NO_3_^−^–N effluent	NH_4_^+^–N removal	NO_2_^−^–N removal	Total nitrogen removal
	influent	effluent	influent	effluent				
*Chloroflexi*	0.18	− 0.30	0.28	− 0.12	0.48*	0.33	0.27	0.27
*Proteobacteria*	− 0.72***	− 0.012	− 0.79***	− 0.048	− 0.69***	− 0.64**	− 0.66**	− 0.67***
*Chlorobi*	0.63***	0.35	0.47*	0.14	0.22	0.29	0.39	0.38
*Planctomycetes*	0.31	0.044	0.48*	0.083	0.49*	0.32	0.36	0.33
*Bacteroidetes*	− 0.21	0.22	− 0.29	0.19	− 0.38	− 0.54*	− 0.57**	− 0.54*
*OD1*	0.74***	0.062	0.72***	0.20	0.43	0.41	0.36	0.43
*Gemmatimonadetes*	− 0.76***	− 0.38	− 0.75***	− 0.44*	− 0.47**	− 0.051	− 0.023	− 0.037
*Armatimonadetes*	0.61**	0.20	0.48*	0.15	0.23	0.23	0.29	0.31
*Actinobacteria*	− 0.71***	0.055	− 0.73***	0.11	− 0.74***	− 0.74***	− 0.79***	− 0.76***
*WS6*	− 0.33	− 0.16	− 0.33	− 0.16	− 0.086	0.16	0.27	0.18
*[Thermi]*	0.28	0.023	0.41	0.24	0.069	0.15	0.085	0.16

*p < 0.05; **p < 0.01; ***p < 0.001

Nearly all bacteria belonging to phylum *Planctomycetes* detected on or after day 38 were anammox bacteria, as detailed below. During the early startup period (days 1–38), relative abundance of *Planctomycetes* remained at a constant low level (~ 1.3%), *Chloroflexi* and *Chlorobi* increased, and *Proteobacteria* and *Bacteroidetes* declined. During the entire running period, *Planctomycetes* abundance was significantly correlated with influent nitrite and effluent nitrate levels (Table 2). Among the phyla with average relative abundance > 1%, *Planctomycetes* showed positive correlation with *Chloroflexi* (*R *= 0.36, *p *< 0.05) and *Acidobacteria* (*R *= 0.38, *p *< 0.05), and negative correlation with *Proteobacteria* (*R *= − 0.69, *p *< 0.001) and *Bacteroidetes* (*R *= − 0.62, *p *< 0.001) (Additional file 1: Fig. S3).

### Bacterial diversity

In the course of the entire running period, ANAMMOX community diversity underwent an initial decrease, then a slight increase, and finally a second decrease to a level at which it remained stable. The Shannon index decreased gradually from 6.87 (day 1) to 4.68 (day 140; minimal value), increased to 6.25 (day 233), then declined to ~ 5.31 and stayed there (Table 1). Pearson’s test between nitrogen concentration (influent ammonia, influent nitrite and effluent nitrite) and α-diversity showed that nitrogen concentration significantly negative correlated to α-diversity (Additional file 1: Table S2).

**Fig. 3:**
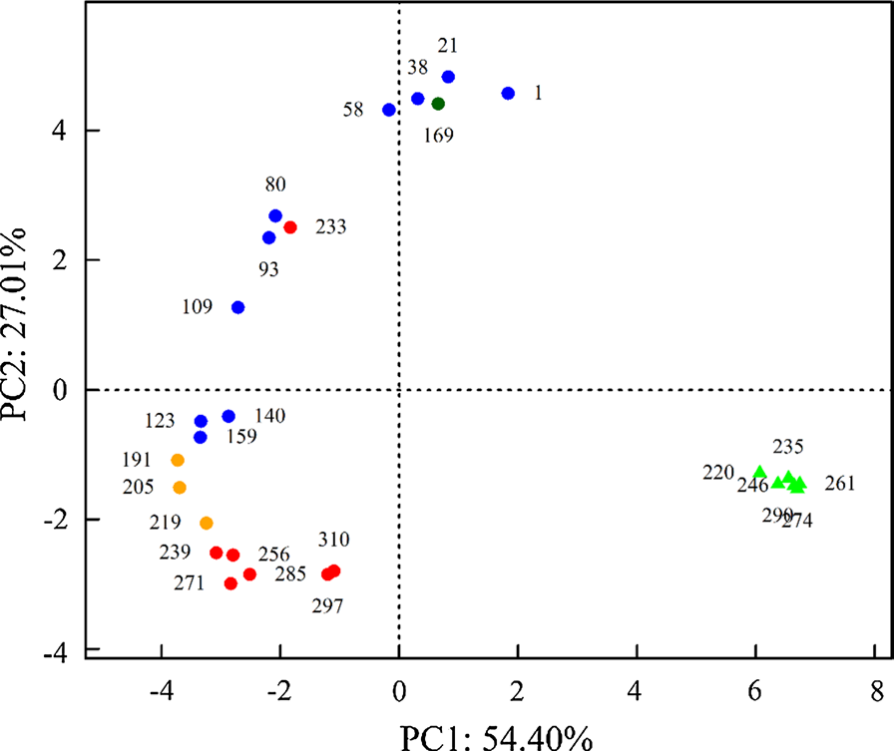
Principal Coordinates Analysis (PCoA) based on Bray–Curtis distance from bacterial 16S rRNA gene sequences. Samples were simply represented by the numbers showing the sampling dates. Triangles: samples from SHARON reactor. Circles: samples from ANAMMOX reactor. Blue: samples obtained with influent load stepwise increased from NH_4_^+^–N 50 mg/L/NO_2_^−^–N 55 mg/L to NH_4_^+^–N 272.7 mg/L/300 mg/L (days 1–160); Bottle-green: samples obtained with influent load NH_4_^+^–N 300 mg/L/NO_2_^−^–N 330 mg/L (days 161–187); Orange: samples obtained with influent load NH_4_^+^–N 272.7 mg/L/NO_2_^−^–N 300 mg/L (days 188–228); Red: samples obtained with SHARON effluent used as influent NH_4_^+^–N ~ 300 mg/L/NO_2_^−^–N ~ 300 mg/L (days 229–310)

In PCoA, the 21 ANAMMOX samples were consistently along the first and second principal coordinates (PC1: 54.40%; PC2: 27.01%), with the exception of samples AN-169 and AN-233 (Fig. 3). The samples generally deviated from sample AN-1 to sample AN-159 gradually along the two PCoA principal coordinates, with stepwise increase of nitrogen load from NH_4_^+^–N 50 mg/L/NO_2_^−^–N 55 mg/L to NH_4_^+^–N 272.7 mg/L/300 mg/L during day 1–160. With nitrogen load increase to NH_4_^+^–N 300 mg/L/NO_2_^−^–N 330 mg/L during day 161–187, sample AN-169 clustered back to AN-1 ~ AN-58. Decrease influent load to NH_4_^+^–N 272.7 mg/L/NO_2_^−^–N 300 mg/L during day 188–228, samples deviated again along the two PCoA principal coordinates following samples AN-123 ~ AN-159. When SHARON effluent was used as ANAMMOX influent during days 229–314, samples deviated a little along the first principal coordinates and clustered together, but AN-233 clustered back to AN-80 and AN-93. The six SHARON samples clustered together, far away from the ANAMMOX samples, consistently with microbial community composition as shown in Fig. 2.

**Fig. 4:**
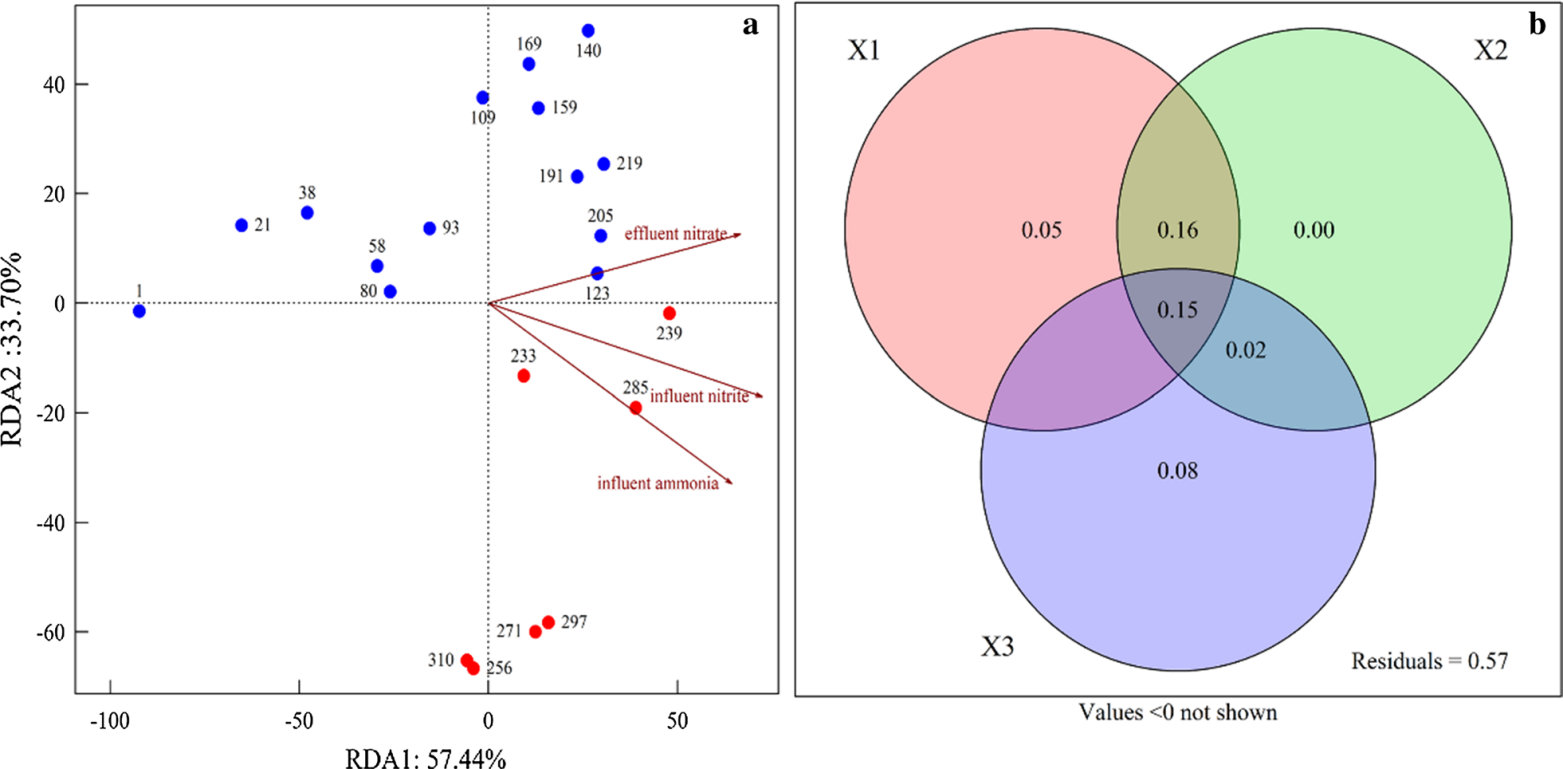
**a** Effects of environmental variables on bacterial community dynamics, from RDA based on Illumina MiSeq data. Numbers: sampling dates. Blue, red, and orange as in Fig. 3. Arrows: direction and magnitude of environmental variables driving bacterial community dynamics. Environmental factors were selected based on significance (*p *< 0.05) calculated from Mantel test results. **b** Relative contributions of environmental variables on bacterial community dynamics, from VPA. X1: influent ammonia; X2: influent nitrite; X3: effluent nitrate

In RDA, influent ammonia (*r*^*2*^= 0.660, *p *< 0.001), influent nitrite (*r*^*2*^= 0.615, *p *< 0.001), and effluent nitrate (*r*^*2*^= 0.523, *p *< 0.01) collectively drove microbial community successions (Fig. 4a). VPA showed that either influent ammonia or influent nitrite explained 33% of community dynamics, whereas effluent nitrate explained 22% (Fig. 4b). These three main parameters collectively contributed to 43% of microbial community dynamics (Fig. 4b), while the remaining 57% was not explained. Other running parameters not shown here, e.g. effluent ammonia, effluent nitrite, pH and DO, contributed little to microbial community dynamics. Mantel test revealed significant correlation between community dynamics and nitrogen removal capability (*r *= 0.489, *p *< 0.001).

### Anammox bacteria and nitrifying bacteria

**Fig. 5:**
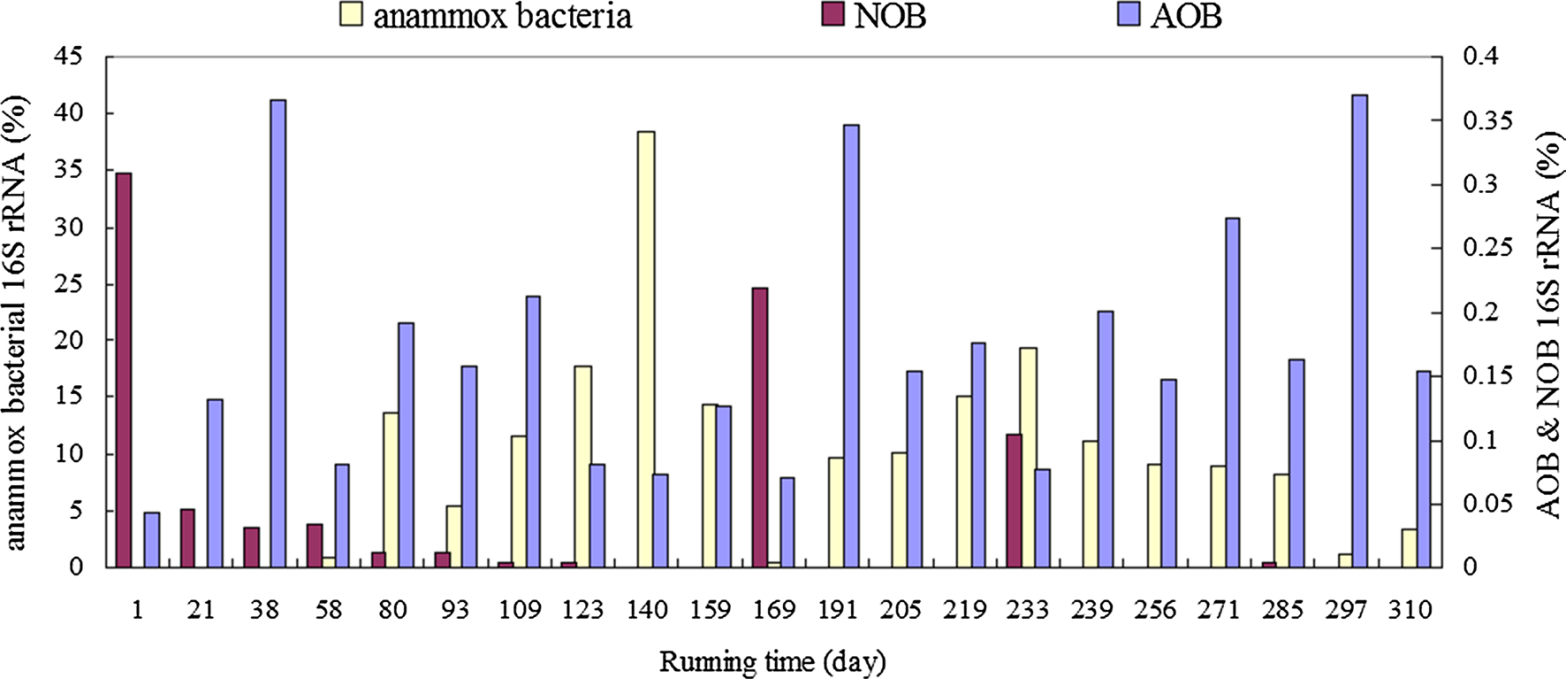
Relative abundance of sequences related to anammox bacteria, NOB, and AOB in ANAMMOX, from bacterial 16S rRNA gene sequences

A heatmap based on annotated genera showed that the two predominant genera in ANAMMOX reactor were *Candidatus Brocadia* and an unclassified genus related to *Ca. Kuenenia* (Fig. S2). Relative abundance of total anammox bacteria became detectable since day 38 with relative abundance 0.03%, increased rapidly to 0.9% on day 58, increased gradually to 38.4% on day 140, decreased to 0.4% on day 169, increased to 19.2% on day 233, remained at ~ 9.0% until day 285, declined to 1.1% on day 297, and then increased to 3.3% on day 310 (Fig. 5). Relative abundance values and variation trends for total anammox bacteria were quite similar to those for *Planctomycetes* as described above.

**Fig. 6:**
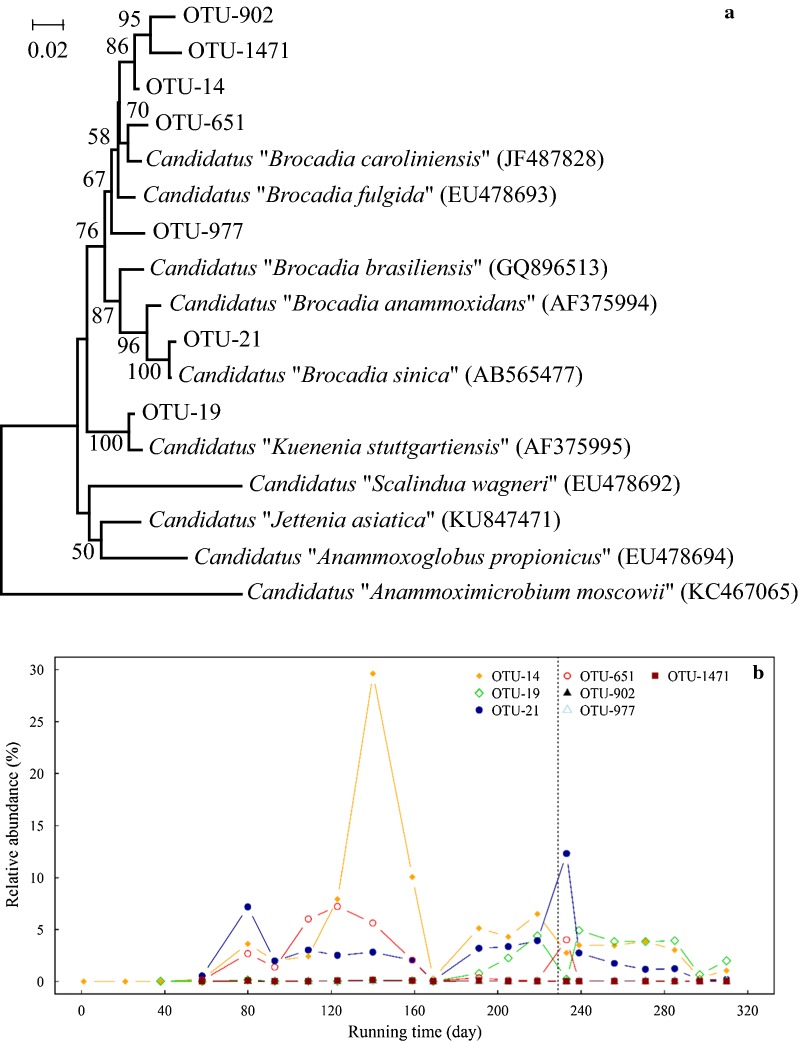
**a** Neighbor-joining tree of anammox bacterial OTUs, based on 16S rRNA gene fragments. Bootstrap values (> 50%) shown on branch nodes are based on 1000 trials. Bar: evolutionary distance 0.01. **b** Relative abundance of these OTUs during the entire running period. Dotted line as in Fig. 1

Seven anammox OTUs in total were defined. A phylogenetic tree was constructed based on these OTUs, which clustered into two groups: a *Ca. Brocadia*-related group (OTU-14, OTU-21, OTU-651, OTU-902, OTU-977 and OTU-1471) and a *Ca. Kuenenia*-related group (OTU-19) (Fig. 6a). The *Ca. Kuenenia*-related group was consistent with the unclassified genus seen in the heatmap (Additional file 1: Fig. S2). During the entire running period, among genera with average relative abundance > 0.05%, *Ca. Brocadia*-related group was positively correlated with genus *Dok59* (*R *= 0.43, *p *< 0.05), which accounted for 0.4–4.8% of 16S rRNA sequences in each sample, but negatively correlated with genera *Gemmatimonas* (*R *= − 0.38, *p *< 0.05) and *Thermomonas* (*R *= − 0.40, *p *< 0.05). *Ca. Kuenenia*-related group was negatively correlated with genera *Dok59* (*R *= − 0.54, *p *< 0.01), *Caldilinea* (*R *= − 0.44, *p *< 0.05), *Bdellovibrio* (*R *= − 0.39, *p *< 0.05), and *Opitutus* (*R *= − 0.41, *p *< 0.05).

Among the seven anammox OTUs, the predominant ones were OTU-14 (97.7% sequence similarity to *Ca*. *Brocadia fulgida*), OTU-19 (98.9% similarity to *Ca*. *Kuenenia stuttgartiensis*), OTU-21 (99.5% similarity to *Ca*. *B. sinica*), and OTU-651 (97.9% similarity to *Ca*. *B. caroliniensis*) (Fig. 6b). The other three OTUs, OTU-902 (95.1% similarity to *Ca. B. fulgida*), OTU-977 (96.5% similarity to *Ca. B. fulgida*), and OTU-1471 (94.8% similarity to *Ca. B. fulgida*) remained at nearly undetectable level throughout the entire running period. In the phylogenetic tree, among *Ca*. *Brocadia*-related group members, OTU-14, -902, and -1471 clustered together to form a subgroup far away from others, and OTU-977 was on a branch by itself.

During the period of increasing influent load (days 1–228), OTU-14 was positively correlated with influent nitrite (*R *= 0.54, *p *< 0.05), and OTU-21 was negatively correlated with effluent ammonia (*R *= − 0.55, *p *< 0.05). OTU-19 and OTU-651 showed no significant correlation with nitrogen compounds. During the subsequent experimental period (days 229–314), the four major OTUs showed no significant correlation with influent or effluent nitrogen compounds.

Relative abundance of the various anammox OTUs underwent dynamic changes. OTU-14, -21, and -651 showed increases from initially undetectable levels during days 1–37, consistently with reactor performance during this period. These three OTUs had similar abundance trends until day 93, dropped greatly on day 169, and showed differing trends thereafter. Abundance of OTU-14 was near zero during days 1–37, increased sharply to 3.6% on day 80, dropped to 2.0% on day 93, increased gradually to 29.6% on day 140, dropped to 0.3% on day 169, increased to 6.5% on day 219, dropped to 0.4% on day 297, and finally increased to 1.1%. Abundance of OTU-21 increased from 0.6% on day 58 to 7.2% on day 80, dropped to ~ 3.0% and stayed there until day 159, dropped further to 0.1% on day 169, increased to 12.3% on day 233, declined gradually to 0.1% on day 297, and then increased slightly to 0.2%. Abundance of OTU-651 increased from near zero to 2.7% during days 1–80, decreased to 1.4% on day 93, increased gradually to 7.2% on day 123, declined to 0.04% on day 169, and remained at nearly undetectable level thereafter, except for a value of 4.0% on day 233. Despite the fluctuations of the three individual OTUs, their combined relative abundance showed an overall increasing trend, consistent with reactor performance during days 1–140 (Fig. 1).

In regard to influent loads (Fig. 1), the three major anammox bacteria as described above were slightly inhibited by differing loads: NH_4_^+^–N 150 mg/L/NO_2_^−^–N 165 mg/L for *Ca. B. sinica* (OTU-21), NH_4_^+^–N 272.7 mg/L/NO_2_^−^–N 300 mg/L for *Ca. B. caroliniensis* (OTU-651), and NH_4_^+^–N 300 mg/L/NO_2_^−^–N 330 mg/L for *Ca. B. fulgida* (OTU-14) (Figs. 1, 5). All three of the above bacteria were greatly inhibited by load NH_4_^+^–N 300 mg/L/NO_2_^−^–N 330 mg/L. On the other hand, *Ca. B. fulgida* and *Ca. B. sinica* recovered from nitrite inhibition after day 169 when influent load dropped, whereas *Ca. B. caroliniensis* did not (Fig. 5). OTU-19, closely related to *Ca. K. stuttgartiensis*, remained at undetectable level during days 1–169, increased to 4.4% on day 219, dropped to 0.2% on day 233, increased to 4.9% on day 239, remained at ~ 4.0% until day 285, dropped to 0.7% on day 297, and then increased to 2.0% on day 310 (Fig. 6b). Trends of relative abundance for OTU-14 and OTU-19 were similar from day 169 onward (*R *= 0.53, *p *< 0.05). SHARON effluent was introduced as ANAMMOX influent on day 229. Resulting decrease of *Ca. B. sinica*-related OTUs was stronger than those of *Ca. B. fulgida*- and *Ca. K. stuttgartiensis*-related OTUs.

Nitrifying bacteria, including ammonia oxidizing bacteria (AOB) and nitrite oxidizing bacteria (NOB), were all detected in the present study (Fig. 5, Additional file 1: Fig. S4). Seven AOB OTUs in total were detected with low relative abundance (0.04–0.36%; Fig. 5), and clustered into three groups: *Nitrosomonas oligotropha*-related group (OTU-831, OTU-1151), *N. ureae*-related group (OTU-1242), and *N. europaea*-related group (OTU-63, OTU-122, OTU-142, OTU-440) (Additional file 1: Fig. S4A). The *N. ureae*- and *N. oligotropha*-related groups were detected respectively during days 1–58 and days 1–80, and remained at undetectable levels thereafter (Additional file 1: Fig. S4B). The *N. europaea*-related group was detected during the entire running period: OTU-122 and -440 during days 1–123, OTU-122 during days 124–219, and OTU-63 and -122 during days 220–310 (Fig. S4B). Four NOB OTUs (OTU-329, -760, -855, -1157) with low relative abundances (0–0.27%, Fig. 5) were detected, with *Nitrospira* being the predominant genus. Relative abundance of total NOB in our study was dynamic, ranging from 0 to 0.27% during the entire running period, e.g., ~ 0.27% on day 1, ~ 0.21% on day 169, and ~ 0.1% on day 233.

## Discussion

To date, little attention has been given to microbial community dynamics in an ANAMMOX reactor including rapid startup, increasing nitrogen load and stable performance (Costa et al. 2014; Liu et al. 2017). A combined system “UASB + SHARON + ANAMMOX” was built for treating piggery wastewater, microbial community dynamics in ANAMMOX reactor were explored using Illumina MiSeq method during the whole running period. Interdependencies of microbial community and running parameters were also elucidated in this study.

### Performance of ANAMMOX reactor

In this study, the ANAMMOX reactor exhibited nitrogen removal ability on day 38, and was successfully started up on day 55; and this performance was well associated with the increase of relative abundance of anammox bacteria. This startup time was much shorter than those in previous reports (e.g., Trigo et al. 2006; Ni and Zhang 2013). This might be due to the shortened doubling time of anammox bacteria with appropriate running parameters designed, e.g. low load, long HRT, appropriate temperature and running manner (Tang et al. 2011). Under such operating conditions, some bacterial taxa increased in relative abundance while others dropped, forming appropriate ecological niches for anammox bacteria (Finlay et al. 1997). Though anammox bacteria were present in the seeding sludge as rare/cryptic microbial species, the nitrogen removal efficiency appeared on day 38. That was because anammox biomass accumulation was necessary to achieve the cell density 10^10^–10^11^ cells mL^−1^, the minimal density required for appreciable nitrogen removal activity (Strous et al. 1999).

Anammox bacteria were the key factor for nitrogen removal performance, and they were affected by influent load dramatically. Anammox bacteria adapted to the increasing nitrogen loads and accumulated its biomass after acclimation, improving nitrogen removal efficiency gradually. However, anammox bacterial growth and nitrogen removal efficiency were adversely inhibited when nitrogen load reached to a certain inhibitory value, and this inhibition could be relieved by reducing influent load to that lower than the inhibitory threshold. The inhibitory effect of nitrite on anammox bacteria is much stronger than that of ammonia (Isaka et al. 2007; Van Hulle et al. 2010). The actual anammox inhibitor is FNA (free nitrous acid) rather than nitrite, and pH greatly affected FNA/nitrite equilibrium (Fernández et al. 2012). An increase of influent NO_2_^−^–N from 55 to 300 mg/L resulted in increase of FNA from ~ 1.7 µg/L to ~ 8.7 µg/L, but the growth of anammox bacteria and reactor performance were not affected too much. However, anammox activity and nitrogen removal efficiency were impaired seriously, resulted from the increased FNA content to ~ 9.6 µg/L with influent NO_2_^−^–N 330 mg/L. Inhibitory nitrite concentration in the present study was lower than the hemi-inhibitory concentration (50% activity loss; IC50) of 11 µg/L reported by Fernández et al. (2012). Anammox performance improved when influent load decreased again to NO_2_^−^–N 300 mg/L, consistently with the findings of Tang et al. (2010). Nitrite in SHARON effluent was NO_2_^−^–N ~ 300 mg/L, but FNA content dropped to ~ 4.0 µg/L due to its high alkaline (pH ~ 8.2). Nitrite inhibition on anammox bacteria could be alleviated for treating pre-treated piggery wastewater.

### Community dynamics

ANAMMOX bacteria, members of *Planctomycetes*, were the functional group in ANAMMOX reactor, and heterotrophic bacteria were also important to ensure its performance (Cho et al. 2010; Finlay et al. 1997), but the microbial community compositions were influenced greatly by influent loads. Increasing influent load stepwise from NH_4_^+^–N 50 mg/L/NO_2_^−^–N 55 mg/L to NH_4_^+^–N 272.7 mg/L/NO_2_^−^–N 300 mg/L (days 1–160), nitrogen removal efficiency were enhanced gradually with the relative abundance of *Planctomycetes*, *Chloroflexi* and *OD1* increased while *Proteobacteria* and *Bacteroidetes* dropped. However, with influent load NH_4_^+^–N 300 mg/L/NO_2_^−^–N 330 mg/L (day 161–187), microbial community compositions were changed greatly, and similar to those during days 1–58. Though nitrogen removal ability was impaired seriously, it was still much higher than that during days 1–58. That was probably because the state of *Planctomycetes* had been changed during the long-period acclimation, and high anammox activity was reserved (Casadesús and D’Ari 2002; Wolf et al. 2010). Reducing the influent load to NH_4_^+^–N 272.7 mg/L/NO_2_^−^–N 300 mg/L (days 188–228), microbial community composition and nitrogen removal rate were similar to those on day 159. With SHARON effluent used as influent since day 229, microbial community and nitrogen removal efficiency on day 233 were similar to those during days 80–93. After acclimation, relative abundance of *Planctomycetes* dropped, but the increase of *Chloroflexi* might enhance the distributed nitrite loop with *Planctomycetes*, promoting the nitrogen removal efficiency (Lawson et al. 2017). These newly formed microbial communities were more suitable for treating pre-treated piggery wastewater.

Relative abundance of *Planctomycetes* was significantly correlated with influent nitrite and effluent nitrate levels (Table 2). Similarly, previous studies showed that nitrite was not only a substrate but also an inhibitor for anammox bacteria (Jin et al. 2012; Strous et al. 1998), and oxidation of nitrite to nitrate supplied reducing power for CO_2_ fixation, as reflected to some degree in bacterial growth (Strous et al. 2006). Notably, its relative abundance dropped greatly to 0.4% on day 169 due to inhibition caused by high nitrite load (NH_4_^+^–N 300 mg/L/NO_2_^−^–N 330 mg/L) (Jin et al. 2012), and this kind inhibition was relieved through reducing influent load (Kimura et al. 2010). *Planctomycetes* showed significant correlation with *Chloroflexi* (*R *= 0.36, *p *< 0.05), *Acidobacteria* (*R *= 0.38, *p *< 0.05), *Proteobacteria* (*R *= − 0.69, *p *< 0.001) and *Bacteroidetes* (*R *= − 0.62, *p *< 0.001) (Additional file 1: Fig. S3), and these microbes helped the formation of a suitable ecological niche for anammox bacteria resulting from constant acclimatization to mineral influent (Finlay et al. 1997). Considering the relationship between these taxa and nitrogen removal efficiency, decline of *Proteobacteria* and *Bacteroidetes* might favor the nitrogen removal efficiency, while *Chloroflexi* and *Acidobacteria* contributed little.

*Proteobacteria* are commonly present in ANAMMOX reactors (Costa et al. 2014; Date et al. 2009; Li et al. 2009). Members of this group are physiologically diverse (including aerobic, anaerobic, microaerobic, and facultatively aerobic forms) and thus able to adapt to a variety of habitats. The oxygen-consuming forms may help create anaerobic environments suitable for anammox bacteria. Members of phylum *Chloroflexi* were reported to feed on lysed anammox bacterial cells and contribute to formation of granular sludge with filamentous structure (Kindaichi et al. 2012; Yamada et al. 2005). Members of *Acidobacteria*, *Bacteroidetes* and *Chlorobi* are always present in ANAMMOX reactors, metabolic activities and interactions between anammox and these heterotrophic bacteria were critical in maintaining the stability of its performance (Lawson et al. 2017).

### Bacterial diversity

Reduced bacterial diversity may result from long-term acclimatization with mineral influent and elimination of unsuitable microorganisms to reach a new equilibrium (Kinnunen et al. 2016). Stepwise increased nitrogen load may open niches for nitrogen metabolism-related organisms, with the microbial community thereby becoming more efficient in nitrogen removal at higher capacity (Finlay et al. 1997). With the period of acclimation in high nitrogen load, microbial diversity and nitrogen removal efficiency on day 169 and 233 quickly recovered, reflecting the strong flexibility of the microbial community in response to external environmental conditions (Casadesús and D’Ari 2002; Finlay et al. 1997). Previous study has revealed that strong effects of ammonia, nitrite, and nitrate levels on anammox community structure (Sun et al. 2014). Community dynamics may also be driven by other influent materials or by environmental factors for the three major running parameters explained 43% of microbial community dynamics (Fig. 4b). Comprehensive evaluation of relationships among microbial community dynamics, nitrogen removal capacity and various environmental conditions will help to determine the optimal running parameters for rapid startup and high efficiency.

### Anammox bacteria, AOB and NOB

Anammox bacteria, only *Ca. Brocadia*-related group and *Ca. Kuenenia*-related group detected, comprised ~ 98.7% of the phylum *Planctomycetes*, similarly to previous findings in the range 87–99.5% (Li et al. 2016). Notably, OTU-14/902/1471 and OTU-977 may constitute two novel species of the genus *Ca. Brocadia*. Their relative abundances were very low during the entire running period, presuming that suitable ecological niches for these taxa were not formed in the ANAMMOX reactor.

Dynamic changes of *Ca. B. fulgida* (OUT-14), *Ca. B. sinica* (OTU-21) and *Ca. B. caroliniensis* (OTU-651) due to stepwise increasing load during days 1–140 suggested that the three anammox bacteria in aerobic sludge attained their ecological niches and adapted to the anaerobic habitat to successfully start up the ANAMMOX reactor (Kinnunen et al. 2016). Considering the impact from influent load, the three major anammox bacteria as described above were slightly inhibited by differing loads (Figs. 1, 6). Numerous studies have shown that ANAMMOX processes are inhibited more strongly by nitrite than by ammonia, but reported threshold values vary widely, from 5 to 280 mg/L NO_2_^−^–N (Isaka et al. 2007; Van Hulle et al. 2010). Community dynamics of anammox bacteria in response to stepwise increasing nitrogen load as observed in the present study may help clarify apparent discrepancies regarding enrichment of various anammox bacteria under differing running conditions (particularly nitrite content), and differing inhibitory nitrite levels for various anammox bacteria. Nitrogen load NH_4_^+^–N 300 mg/L/NO_2_^−^–N 330 mg/L was the maximum inhibitory concentration, in agreement with the previous report that NO_2_^−^–N 280 mg/L should be considered a warning value (Jin et al. 2012). The nitrite inhibition was relieved when influent load dropped, which was consistent with previous research (Kimura et al. 2010), but not for all anammox bacteria in this study.

*Ca. K. stuttgartiensis* (OTU-19) was detected and increased since day 169 (Fig. 6b). An ecological niche defined by high nitrogen load may be necessary for growth of OTU-19, since *Ca. K. stuttgartiensis* was frequently detected in ANAMMOX reactors with high nitrogen load in previous studies (Li et al. 2009; Wang et al. 2013). Relative abundance for OTU-14 and OTU-19 were correlated from day 169 onward (*R *= 0.53, *p *< 0.05), suggesting that their ecological niches and/or physiological functions are similar, and differ from those of *Ca. B. sinica*. *Ca. B. fulgida* and *Ca. K. stuttgartiensis* both responded to iron accumulation in ANAMMOX reactor, which was reported previously to be a stimulatory factor for growth of anammox bacteria (van Niftrik et al. 2008; Zhang et al. 2009).

Total relative abundance of anammox bacteria dropped to < 10% and OTU relative abundance fluctuated greatly when using SHARON effluent since day 229. Interestingly, nitrogen removal rate was barely affected after acclimation, resulting from the higher anammox activities of *Ca. B. fulgida*, *Ca. B. sinica* and *Ca. K. stuttgartiensis* besides the distributed nitrite loop described above. Decrease of *Ca. B. sinica*-related OTUs was stronger than those of *Ca. B. fulgida*- and *Ca. K. stuttgartiensis*-related OTUs, speculating that *Ca. B. sinica* has greater sensitivity to SHARON effluent. Similar relative abundance of anammox bacteria was also detected previously when ANAMMOX reactors were fed with pre-treated wastewater from partial nitrification [7.9%, Dosta et al. (2015); 0.3%, Wang et al. (2016)]. The decline of relative abundance of anammox bacteria might be due to the ambiguous compositions in piggery wastewater, e.g., antibiotics, heavy metals, phosphate and sulfide (Bernet et al. 2000; Tang et al. 2011; Tong et al. 2009; Jin et al. 2012). Previous studies demonstrated the complexity of components in SHARON effluent, and sensitivity of anammox activity to external interference at high influent load (Fernández et al. 2012; Krümmel and Harms, 1982). However, such external interference is presumably not due to organic materials, since they are removed efficiently during UASB and SHARON processes, as indicated by very low COD content of SHARON effluent (data not shown). Values of DO and pH in SHAORN effluent (0.03–0.16 mg/L; ~ 8.2) were both under the inhibition threshold of anammox bacteria according to Egli et al. (2001) and Strous et al. (1999).

AOB and NOB are commonly present in ANAMMOX reactors (Kindaichi et al. 2012; Li et al. 2009; Liu et al. 2017; Park et al. 2010). Relative abundance of total AOB was low (0.04–0.36%; Fig. 5), less than the 1.78% value reported by Liu et al. (2017). AOB groups in the present study were diverse and variable, including *N. europaea*, *N. ureae* and *N*. *oligotropha*, whereas previous studies found *N. europaea* to be the predominant AOB (Liu et al. 2017; Pereira et al. 2017). Genus *Nitrospira*, the predominant NOB, was detected with dynamic changes in low relative abundances (0–0.27%, Fig. 5). Previous studies found *Nitrospira* to be the predominant NOB in ANAMMOX reactors, with relative abundance averaging ~ 3.8% (Kindaichi et al. 2012; Li et al. 2009; Liu et al. 2017; Park et al. 2010)—a considerably higher value than that in the present study.

In conclusion, we explored the microbial community dynamics in an ANAMMOX reactor from startup, increasing nitrogen load and stable performance for piggery wastewater treatment, which filled the gap among microbial community composition/anammox bacteria, running parameters and bioreactor performance. Microbial community composition and functional taxa played important roles in nitrogen removal efficiency. Influent ammonia, influent nitrite and effluent nitrate drove the dynamics of microbial community greatly, and some undefined factors, might also influence this dynamics, needed further study. Anammox bacteria could be enriched or eliminated depending on their ecological niches formed or not. Nitrite inhibition values to anammox groups were also elucidated, and this not only could explain the controversy of nitrite inhibition values but also revealed the optimal nitrogen loads to different anammox groups. These results would be useful to optimize running parameters for fast startup and high, stable efficiency of such bioreactors in practical application.

## Additional file

**Additional file 1: Figure S1.** Schematic diagram of the “UASB+SHARON+ANAMMOX” system for piggery wastewater treatment. (1) storage tank; (2) delivery pump; (3) gas meter; (4) UASB reactor; (5) effluent tank; (6) delivery pump; (7) warm water delivery pump; (8) SHARON reactor; (9) thermostat water bath; (10) air pump; (11) effluent tank; (12) suction pump; (13) delivery pump; (14) ANAMMOX reactor; (15) warm water delivery pump; (16) effluent tank. **Figure S2.** Heatmap plot illustrating relative percentages of major genera (clustering shown on vertical axis) within each sample (horizon-axis clustering) from ANAMMOX bioreactor. Numbers at bottom: times when activated sludge samples were obtained. Color intensities indicate relative abundances at genus level (legend at bottom).** Figure S3.** Four phyla were significantly correlated to Planctomycetes in the ANAMMOX bioreactor.** Figure S4.** A) Neighbor-joining tree of AOB OTUs based on 16S rRNA gene fragments. B) Relative abundance of these OTUs during the entire experimental period. Dotted line indicated day 229 when effluent from SHARON was used as influent. Bootstrap values (>50%) shown on branch nodes are based on 1000 trials. Bar: evolutionary distance 0.01.

## Abbreviations

UASB: Upflow Anaerobic Sludge Blanket
SHARON: Single reactor system for High activity Ammonium Removal Over Nitrite
ANAMMOX: anaerobic ammonium oxidation
HRT: hydraulic retention time
COD: chemical oxygen demand
DO: dissolved oxygen
VS: volatile solids
SS: suspended solids
QIIME: Quantitative Insights Into Microbial Ecology
OTU: operational taxonomic units
PCoA: principle coordinates analysis
RDA: redundancy analysis
VPA: variation partitioning analysis
NJ: neighbor-joining
