# Supplementary material for: Microbial community dynamics in an ANAMMOX reactor for piggery wastewater treatment with startup, raising nitrogen load, and stable performance
Source: AMB Express. 2018 Oct 1;8:156. doi: 10.1186/s13568-018-0686-0 (PMC6167272; doi:10.1186/s13568-018-0686-0)

# Submitted to AMB Express

# Additional file for:

**Microbial community dynamics in an ANAMMOX reactor for piggery wastewater treatment with startup, raising nitrogen load, and stable performance**

Qiang Huang^1,2^, Wei-Li Du^1,2^, Li-Li Miao^1^, Ying Liu^1^, Zhi-Pei Liu^1*^

1 State Key Laboratory of Microbial Resources, Institute of Microbiology, Chinese Academy of Sciences, Beijing 100101, P. R. China.

2 University of Chinese Academy of Sciences, Beijing 100049, P.R. China.

*Corresponding author. Phone: (86) 64806081; e-mail: liuzhp@sun.im.ac.cn

**Additional Information**

**Legends to Additional Figures**

**Figure S1**. Schematic diagram of the “UASB+SHARON+ANAMMOX” system for piggery wastewater treatment. (1) storage tank; (2) delivery pump; (3) gas meter; (4) UASB reactor; (5) effluent tank; (6) delivery pump; (7) warm water delivery pump; (8) SHARON reactor; (9) thermostat water bath; (10) air pump; (11) effluent tank; (12) suction pump; (13) delivery pump; (14) ANAMMOX reactor; (15) warm water delivery pump; (16) effluent tank.

**Figure S2**. Heatmap plot illustrating relative percentages of major genera (clustering shown on vertical axis) within each sample (horizon-axis clustering) from ANAMMOX bioreactor. Numbers at bottom: times when activated sludge samples were obtained. Color intensities indicate relative abundances at genus level (legend at bottom).

**Figure S3**. Four phyla were significantly correlated to *Planctomycetes* in the ANAMMOX bioreactor.

**Figure S4.** A) Neighbor-joining tree of AOB OTUs based on 16S rRNA gene fragments. B) Relative abundance of these OTUs during the entire experimental period. Dotted line indicated day 229 when effluent from SHARON was used as influent. Bootstrap values (>50%) shown on branch nodes are based on 1000 trials. Bar: evolutionary distance 0.01.

**Table S1**. Parameters of component reactors of “UASB+SHARON+ANAMMOX” system for piggery wastewater treatment.

| Reactor | UASB | SHARON | ANAMMOX |
| --- | --- | --- | --- |
| Height/diameter  Effective volume (L)  Temperature (ºC)  Hydraulic retention time (h) | 12/1  13.3  31-32  26.6 | 10/3  12.5  room temperature  25 | 12/1  13.3  31-32  26.6 |

**Table S2 Correlations (*R* values) between** **α-diversity indices of microbial communities and running parameters by Pearson's test.**

| Indexes | NH_4_^+^-influent | NH_4_^+^-effluent | NO_2_^-^-influent | NO_2_^-^-effluent | NO_3_^-^-effluent | NH_4_^+^- removal | NO_2_^-^- removal | TN- removal |
| --- | --- | --- | --- | --- | --- | --- | --- | --- |
| ACE  chao1  shannon  simpson | -0.48*  -0.49*  -0.69***  -0.46* | 0.33  0.29  0.046  0.047 | -0.55*  -0.56**  -0.73***  -0.55* | 0.14  0.15  -0.061  -0.094 | -0.66**  -0.67***  -0.61**  -0.54* | -0.59**  -0.60**  -0.55*  -0.37 | -0.49*  -0.53*  -0.51*  -0.34 | -0.54*  -0.56**  -0.55**  -0.35 |

* *p*< 0.05, ** *p*< 0.01, *** *p*< 0.001.

**Figure S1**


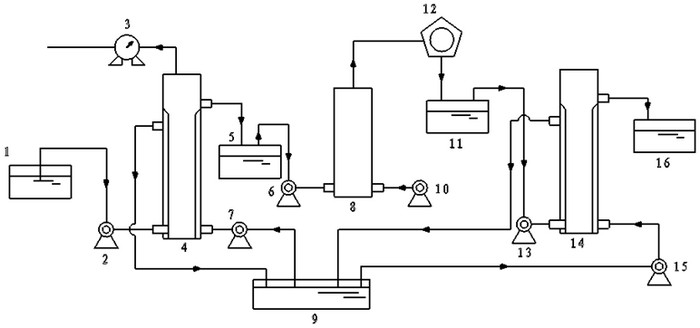


**Figure S2**





**Figure S3**


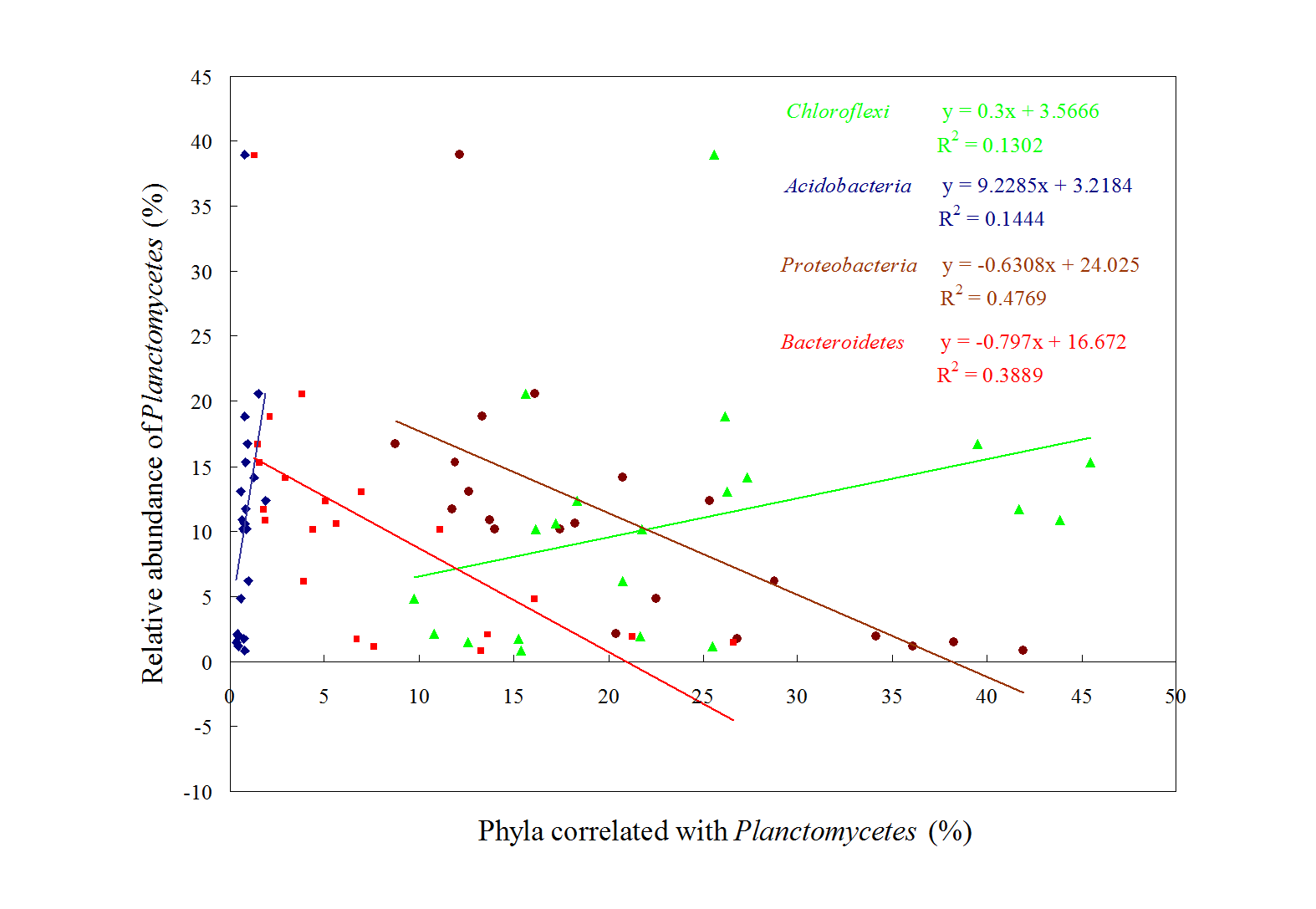


**Figure S4**

A


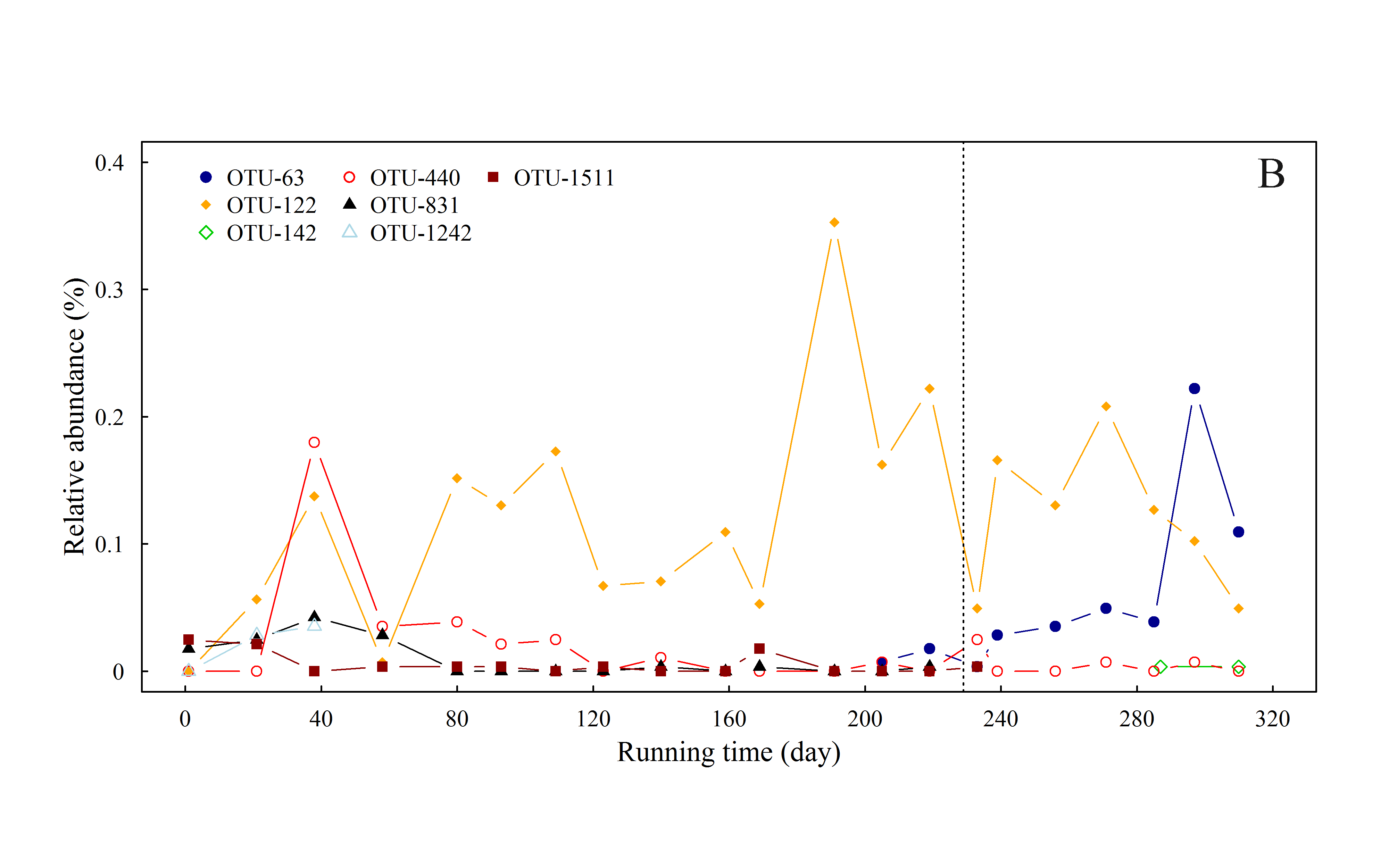

Supplement: Supplementary file 1 — Additional file 1: Figure S1. Schematic diagram of the “UASB+SHARON+ANAMMOX” system for piggery wastewater treatment. (1) storage tank; (2) delivery pump; (3) gas meter; (4) UASB reactor; (5) effluent tank; (6) delivery pump; (7) warm water delivery pump; (8) SHARON reactor; (9) thermostat water bath; (10) air pump; (11) effluent tank; (12) suction pump; (13) delivery pump; (14) ANAMMOX reactor; (15) warm water delivery pump; (16) effluent tank. Figure S2. Heatmap plot illustrating relative percentages of major genera (clustering shown on vertical axis) within each sample (horizon-axis clustering) from ANAMMOX bioreactor. Numbers at bottom: times when activated sludge samples were obtained. Color intensities indicate relative abundances at genus level (legend at bottom). Figure S3. Four phyla were significantly correlated to Planctomycetes in the ANAMMOX bioreactor. Figure S4. A) Neighbor-joining tree of AOB OTUs based on 16S rRNA gene fragments. B) Relative abundance of these OTUs during the entire experimental period. Dotted line indicated day 229 when effluent from SHARON was used as influent. Bootstrap values (>50%) shown on branch nodes are based on 1000 trials. Bar: evolutionary distance 0.01. [file 13568_2018_686_MOESM1_ESM.doc]
